# Identification of nuclear valosin-containing-protein-like as a target of anti-nuclear autoantibodies in systemic sclerosis

**DOI:** 10.3389/fmed.2024.1477365

**Published:** 2025-01-21

**Authors:** Zitao Zeng, Ramona Miske, Madeleine Scharf, Yvonne Denno, Anthonina Ott, Stefanie Brakopp, Bianca Teegen, Winfried Stöcker, Elise Siegert, Sandra Saschenbrecker, Christian Probst, Lars Komorowski

**Affiliations:** ^1^Institute for Experimental Immunology, affiliated to EUROIMMUN Medizinische Labordiagnostika AG, Lübeck, Germany; ^2^Clinical Immunological Laboratory, Groß Grönau, Germany; ^3^Department of Rheumatology and Clinical Immunology, Charité – Universitätsmedizin Berlin, Berlin, Germany; ^4^Berlin Institute of Health at Charité - Universitätsmedizin Berlin, Berlin, Germany

**Keywords:** ANA, anti-nuclear autoantibodies, autoimmune disease, biomarker, diagnosis, nuclear valosin-containing-protein-like, NVL, systemic sclerosis

## Abstract

**Objective:**

To identify the target antigen of an anti-nuclear autoantibody (ANA) from a patient with a suspected systemic autoimmune disease and to study the autoantibody’s clinical association.

**Methods:**

The index patient serum was screened for autoantibodies using indirect immunofluorescence assay (IFA) and line blots (membrane strips coated with parallel lines of different purified antigens). Immunoprecipitation with fixed HEp-2 cells followed by SDS-PAGE and MALDI-TOF mass spectrometry was used to identify the autoantigen, which was verified by competitive inhibition experiments, recombinant HEK293 cell-based IFA, and Western and line blots based on the recombinant antigen. The prevalence of autoantibodies against this antigen was studied in 693 patients with systemic autoimmune rheumatic diseases (SARD) and 150 healthy controls.

**Results:**

The index patient serum displayed a homogeneous nucleolar staining pattern on HEp-2 cells and monkey liver by IFA but did not react with 27 known nuclear antigens. Nuclear valosin-containing-protein-like (NVL) was identified as the ANA target antigen. Preincubation with recombinant NVL abolished the reactivity of the patient serum with HEp-2 cells in IFA. Additionally, the patient serum reacted with recombinant NVL in cell-based IFA and Western blot analysis, whereas sera from 15 healthy controls were nonreactive. Using line blots coated with recombinant NVL, anti-NVL autoantibodies were exclusively found in four out of 378 patients with systemic sclerosis, but neither in 315 patients with other SARD nor in 150 healthy controls.

**Conclusion:**

These findings indicate that autoantibodies against NVL may be a suitable marker to help narrowing the serological gap in systemic sclerosis.

## 1 Introduction

Anti-nuclear autoantibodies (ANA) are important biomarkers of systemic autoimmune rheumatic diseases (SARD), also referred to as connective tissue diseases. This heterogeneous group of disorders includes, among others, systemic lupus erythematosus, Sjögren’s syndrome, systemic sclerosis (SSc), polymyositis/dermatomyositis and mixed connective tissue disease, with ANA evidenced in 20–100% of cases (1). Among various clinical, laboratory, imaging and pathology parameters relevant for the classification of SARD, differential ANA diagnostics is crucial for the identification of several rheumatic diseases and for accurate clinical decision-making. In some cases, the presence and titer of these autoantibodies (AAb) can also help to assess prognosis and to monitor disease activity, progression or treatment response.

For example, SSc is associated with three major disease-specific AAb, namely anti-DNA topoisomerase I (anti-TOPO-1), anti-centromere (ACA), and anti-RNA polymerase III (anti-RNAP III). These AAb are associated with distinct clinical phenotypes within the SSc spectrum: ACA-positive patients often exhibit mild manifestations, anti-TOPO-1-positive individuals are more prone to develop interstitial lung disease, whereas the presence of anti-RNAP III indicates an increased risk of diffuse disease, scleroderma renal crisis and cancer. Besides, there are other AAb (e.g., anti-Ku, anti-PM-Scl) which are present in overlap syndromes such as polymyositis/SSc (2, 3).

For AAb screening in SARD, the indirect immunofluorescence assay (IFA) on human epithelial type 2 (HEp-2) cells is considered as gold standard. This cell substrate provides a larger target composition than other screening assays, allowing the determination of AAb directed against multiple intra-cellular antigens, including nuclear, cytoplasmic and mitotic apparatus components (4). Provided there is a strong clinical suspicion of an underlying connective tissue disease, a positive (or ambiguous) ANA IFA result is usually further examined for the targeted autoantigen. Depending on the ANA pattern feature and on clinical information, appropriate antigen-specific tests (e.g., ELISA, immunoblot) are chosen for further specification of ANA subtypes.

Over the last decade, there has been significant progress in the characterization of so far unidentified ANA antigens. Nevertheless, there is still a small subset of SARD patients with unknown ANA reactivity. The aim of this work was to identify the ANA target in a patient suspected of having a systemic autoimmune disease and to study the clinical association.

## 2 Materials and methods

Reagents were obtained from Merck (Darmstadt, Germany) or Sigma-Aldrich (Heidelberg, Germany) if not specified otherwise.

### 2.1 Patients

The Clinical Immunological Laboratory Stöcker (Lübeck, Germany) received the serum samples for the purpose of AAb testing. An anonymized serum sample showing a nucleolar ANA pattern but with negative results in the requested antigen-specific tests was provided to the authors for the purpose of AAb identification. Clinical data were not available for this index patient (80 years, female).

In addition, serum samples were obtained from patients with SARD, including 378 SSc patients (5) and 315 patients with other diseases (autoimmune hepatitis, *n* = 40; anti-synthetase syndrome, *n* = 10; inclusion body myositis, *n* = 4; mixed connective tissue disease, *n* = 6; myositis, *n* = 15; primary Sjögren’s syndrome, *n* = 11; rheumatoid arthritis, *n* = 54; systemic lupus erythematosus, *n* = 165; undifferentiated connective tissue disease, *n* = 10). Sera from 150 healthy donors were tested as controls. Most samples were collected at the Department of Rheumatology and Clinical Immunology, Charité – Universitätsmedizin Berlin (Berlin, Germany); the remaining samples were obtained (anonymized) from commercial sources in accordance with ethical standards. Demographics and diagnostic criteria of the study population are given in Supplementary Table S1.

### 2.2 Recombinant synthesis of target antigen and purification

Cloning and expression of nuclear valosin-containing-protein-like (NVL) in *E. coli* and in HEK293 as well as protein purification were conducted as in Radzimski et al. (6). Briefly, the cDNA clone IRAUp969F0866D encoding a partial sequence (amino acids 206–856) of human NVL-isoform1 (UniProt acc. no. O15381-1) was obtained from Source BioScience United Kingdom Limited (Nottingham, United Kingdom). This cDNA clone is coding NVL-isoform3 (UniProt acc. no. O15381-3, GenBank acc. no. BC012105). A synthetic DNA fragment coding for the missing amino acids 1–205 of NVL-isoform1 was ordered from Eurofins Genomics Germany GmbH (Ebersberg, Germany). The gene synthesis product was digested with BsaI (R3733, New England Biolabs, Frankfurt am Main, Germany) and the 614 bp fragment was isolated by gel purification. The coding sequence for the amino acid 206–856 of NVL-isoform1 was amplified by PCR using cDNA clone IRAUp969F0866D and DNA oligonucleotide primers sense NVL-IF1 (ataggtctccaggattcaaaagattcttc) and asense NVL-IF1 (tatggtctcatcgacccggc-tgagggactcctgc). The amplification product was digested with BsaI and together with the gene synthesis fragment ligated with NcoI/XhoI-linearized pTriEx-1 or pET24d vector, adding octahistidine- or hexahistidine-tag to the C-terminus.

Using these vectors, recombinant full-length NVL (amino acids 1–856) was expressed in *E. coli* Rosetta (DE3) pLacI (Novagen, Darmstadt, Germany) and purified under denaturing conditions (50 mM sodium phosphate pH 7.4, 1 M NaCl, 8 M urea). Additionally, the protein was expressed in HEK293 cells for IFA as previously described by Radzimski et al. (6).

### 2.3 Indirect immunofluorescence assay

IFA was conducted as in Scharf et al. (7). Briefly, slides with a biochip mosaic of acetone-fixed HEp-2 cells combined with liver tissue cryosections, or recombinant human embryonic kidney (HEK) 293 cells expressing NVL, and control-transfected HEK293 cells were used for IFA (EUROIMMUN Medizinische Labordiagnostika AG, Lübeck, Germany). After serum incubation, fluorescein isothiocyanate-labeled goat anti-human IgG-Fc (EUROIMMUN) or Alexa488-labeled anti-human IgG-Fc (Jackson Research, Suffolk, United Kingdom) were applied. In competitive inhibition experiments, the purified recombinant full-length human NVL antigen (see section 2.2) or preparation/control buffer (50 mM sodium phosphate pH 7.4, 1 M NaCl, 8 M urea) was mixed (final dilution 1:10) with the patient serum (final dilution 1:1000) in phosphate-buffered saline (PBS)-Tween (1x PBS pH 7.2, 0.2% Tween-20) 1 h prior to the IFA testing on HEp-2 cells. Results were evaluated by two independent observers using a EUROStar II fluorescence microscope (EUROIMMUN). A titer of 1:100 was used as cut-off for ANA positivity on cell- and tissue-based substrates.

### 2.4 Immunoprecipitation and identification of candidate antigens

Glass coverslips coated with acetone-fixed HEp-2 cells (EUROIMMUN) were incubated with patient serum at a dilution of 1:200 at 4°C for 3 h. After 3 washes with PBS, immune complexes were solubilized in buffer (1x PBS pH 7.4, 0.1% (v/v) NP40, 1% (w/v) deoxycholate, 0.1% (w/v) sodium dodecyl sulfate (SDS) including 1 mM protease inhibitor cocktail). Detached material was homogenized and centrifuged at 16,000 g at 4°C for 20 min. IgG and immune complexes in supernatant were then immobilized on Protein G Dynabeads (ThermoFisher Scientific, Schwerte, Germany) overnight at 4°C. After washing 3 times with PBS, the beads were eluted with lithium dodecyl sulfate (LDS) sample buffer (NuPAGE, Thermo Fisher Scientific) containing 25 mmol/L dithiothreitol and iodoacetamide at 70°C for 10 min. SDS-polyacrylamide gel electrophoresis (SDS-PAGE) and mass spectrometry were applied for protein identification.

### 2.5 Mass spectrometry

Sample preparation for matrix-assisted laser desorption/ionization-time of flight (MALDI-TOF) mass spectrometry was conducted as previously described (8). Hardware, software, MALDI targets, peptide standards and matrix reagents were from Bruker Daltonics (Bremen, Germany), unless otherwise noted. In brief, after SDS-PAGE and staining of the gel with Coomassie Brilliant Blue G-250, the protein band of interest was excised and destained. Following tryptic digestion, the peptides were extracted and spotted with *α*-cyano-4-hydroxycinnamic acid onto a MTP AnchorChip 384 TF target. MALDI-TOF analysis was carried out on an Autoflex III Smartbeam TOF/TOF200 mass spectrometer controlled by flexControl software v.3.4. For peptide mass fingerprinting (PMF), the mass spectrum was acquired in the positive ion reflector mode 6,000 shots, mass range 600–4,000 Da. The spectrum was calibrated with Peptide Calibration Standard II and the raw data were processed into a peak list using flexAnalysis software v.3.4. Processed data were analyzed with BioTools v.3.2 using the Mascot Server v.2.3 (Matrix Science, London, United Kingdom) for protein identification by searching against the NCBI or SwissProt database. Search was conducted with the following parameters: taxonomy: *Homo sapiens*, mass tolerance: 80 ppm, maximum 1 missed cleavage, fixed modification: cysteine carbamidomethylation, variable modification: methionine oxidation, significance threshold: *p* < 0.05.

### 2.6 SDS-PAGE and Western blot

Purified recombinant NVL was separated by SDS-PAGE and electrotransferred onto a nitrocellulose membrane by tank blotting with transfer buffer (ThermoFisher Scientific) according to the manufacturer’s instructions. The membranes were blocked with Universal Blot Buffer plus (EUROIMMUN) for 15 min and incubated with human serum or murine anti-His tag antibodies in Universal Blot Buffer plus for 3 h, followed by 3 washing steps with Universal Blot Buffer (EUROIMMUN). After a second incubation with anti-human-IgG-Fc-alkaline phosphatase (EUROIMMUN) or anti-mouse-IgG-alkaline phosphatase (Jackson Research), the membranes were washed 3 times with Universal Blot Buffer and stained with nitro blue tetrazolium/5-bromo-4-chloro-3-indolyl phosphate (NBT/BCIP) substrate (EUROIMMUN).

### 2.7 Line blot

Line blots are membrane-based enzyme immunoassays for the highly specific determination of antibodies in patient samples. The test strips consist of several membrane chips which are fixed at defined positions on a plastic foil strip. Each membrane chip is coated with thin parallel lines of highly purified, biochemically characterized antigens. The combination of multiple antigens on one test strip enables simultaneous screening for different disease-relevant antibodies. If a sample contains specific antibodies, these bind to the corresponding immobilized antigen. To detect the bound antibodies, enzyme-labeled anti-human IgG is added. This enzyme conjugate catalyzes a color reaction after addition of a substrate solution, resulting in a dark line at the respective antigen position. Control bands on each test strip indicate whether the individual incubation steps have been performed correctly.

The EUROLINE Systemic Sclerosis (Nucleoli) Profile (IgG), EUROLINE ANA Profile 23 (IgG) and a customized research line blot with purified recombinant NVL were employed in this study (all assays from EUROIMMUN). They were incubated according to the manufacturer’s instructions for use and analyzed using the software EUROLineScan (EUROIMMUN).

### 2.8 Statistics

Confidence intervals [95% CI] were calculated using the method of Clopper and Pearson. Statistical analyses were conducted using GraphPad Prism v.10.3.1 (GraphPad Software, Inc., San Diego, CA, United States).

## 3 Results

### 3.1 Characterization of patient serum and identification of NVL

**Figure 1:**
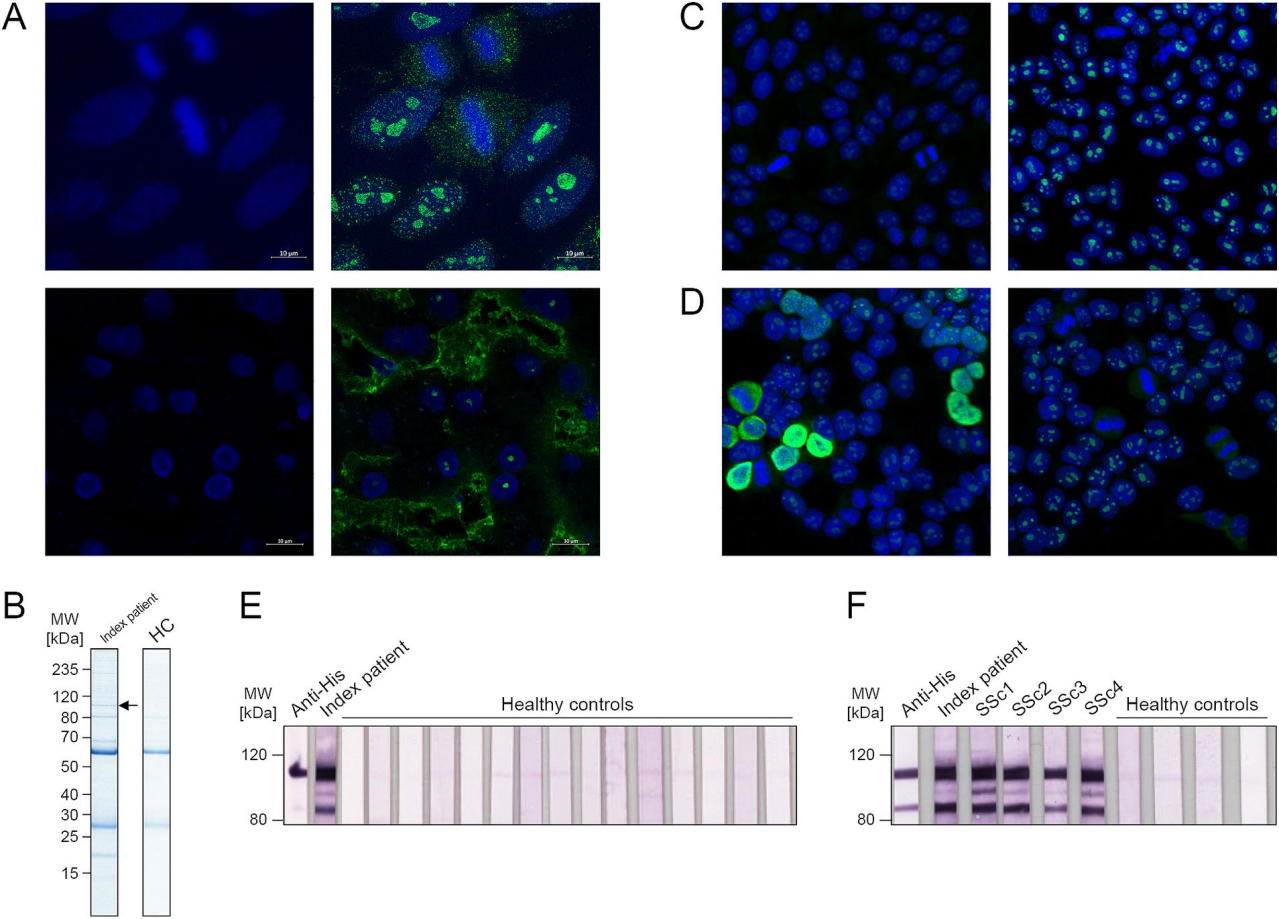
Identification and verification of nuclear valosin-containing-protein-like (NVL) as target of anti-nuclear autoantibodies. **(A)** Indirect immunofluorescence assay (IFA) with index patient serum (1:1000) on HEp-2 cells (top) and monkey liver (bottom), stained with anti-human-IgG-Alexa488 (1: 500, green) as secondary antibody and To-Pro-3 (1:2000, blue) as nuclear counterstain. Shown are single channel images (left, DNA only) and merged two-color images (right, DNA and patient serum). Magnification 630x. **(B)** Immunoprecipitates of serum from index patient or healthy control (HC) and HEp-2 cell lysate, stained with Coomassie after SDS-PAGE. The marked band was selected for mass spectrometry analysis. **(C)** Competitive inhibition experiments. Index patient serum (1:1000) was incubated with the 1:10 diluted purified recombinant NVL antigen (left) or control buffer (right) prior to application in IFA with HEp-2 cells. Anti-Human-IgG-Alexa488 (1: 500) was used as secondary antibody and To-Pro-3 (1:2000) was applied as nuclear counterstain. Shown are merged two-color images. Magnification 200x. **(D)** IFA with index patient serum (1:1000) on NVL-transfected (left) or control-transfected (right) HEK293 cells. Anti-Human-IgG-Alexa488 (1: 500) was used as secondary antibody and To-Pro-3 (1:2000) was applied as nuclear counterstain. Shown are merged two-color images. Magnification 200x. **(E,F)** Western blot of purified His-tagged NVL antigen incubated with anti-His tag antibody (1:2000), index patient serum (1:200), 15 sera from healthy controls (1:200) **(E)** or sera (1:200) from anti-NVL-positive patients with systemic sclerosis (SSc) **(F)**. Anti-mouse-IgG-AP (1:2000) or anti-human-IgG-B/E-AP (1:10) were used in secondary incubation.

The index patient serum revealed a homogenous nucleolar pattern (titer 1:3,200) in IFA using HEp-2 cells and monkey liver (AC-8 according to the International Consensus on ANA Patterns [ICAP] (9)), as presented in Figure 1A. Line blots coated with 27 known ANA target antigens (dsDNA, nucleosomes, histones, SSA, Ro-52, SSB, RNP/Sm, Sm, Mi-2α, Mi-2β, Ku, CENP A, CENP B, Sp100, PML, Scl-70, PM-Scl100, PM-Scl75, RP11, RP155, gp210, PCNA, fibrillarin, NOR90, Th/To, PDGFR and DSF70) showed no positive reactions with the index patient serum.

Immunocomplexes precipitated with index patient serum from HEp-2 cells were resolved by SDS-PAGE and stained with Coomassie blue, revealing a protein band of approximately 100 kDa (Figure 1B), which was absent in immunoprecipitation eluates of healthy control sera. Western blots of HEp-2 cell lysates showed only the 100 kDa band after incubation with the index patient serum (data not shown). Therefore, this band was excised for MALDI-TOF mass spectrometry analysis and PMF. The protein was identified as human nuclear valosin-containing protein-like (NVL, UniProt acc. no. O15381) with a molecular weight of 96 kDa, a Mascot score of 164 and a 33% sequence coverage (Supplementary Table S2).

### 3.2 Verification of NVL as target antigen

Human NVL was recombinantly expressed in *E. coli* and the purified antigen was applied in competitive inhibition experiments. The fluorescence pattern of the index patient serum on HEp-2 cells was abolished when the serum was preincubated with NVL but remained unchanged after preincubation with control buffer (Figure 1C), indicating a relation between the observed nucleolar pattern and the AAb against NVL. In addition, incubation of the index patient serum with HEK293 cells expressing recombinant NVL revealed a specific nuclear fluorescence pattern. This pattern, however, could not be observed after incubation with control-transfected HEK293 cells (which showed only the nucleolar staining of endogenous NVL), confirming the presence of anti-NVL AAb in the index patient serum (Figure 1D). Furthermore, when the purified recombinant antigen was applied in Western blot, positive reactions were only found after testing with anti-His tag antibody and index patient serum, whereas 15 sera from healthy controls showed no comparable reaction (Figure 1E). This Western blot analysis revealed not only an NVL band at 100 kDa, but also an additional band with an apparent molecular weight of about 80 kDa, possibly indicating the presence of an N-terminally truncated form of NVL.

### 3.3 Clinical association of anti-NVL autoantibodies

**Figure 2:**
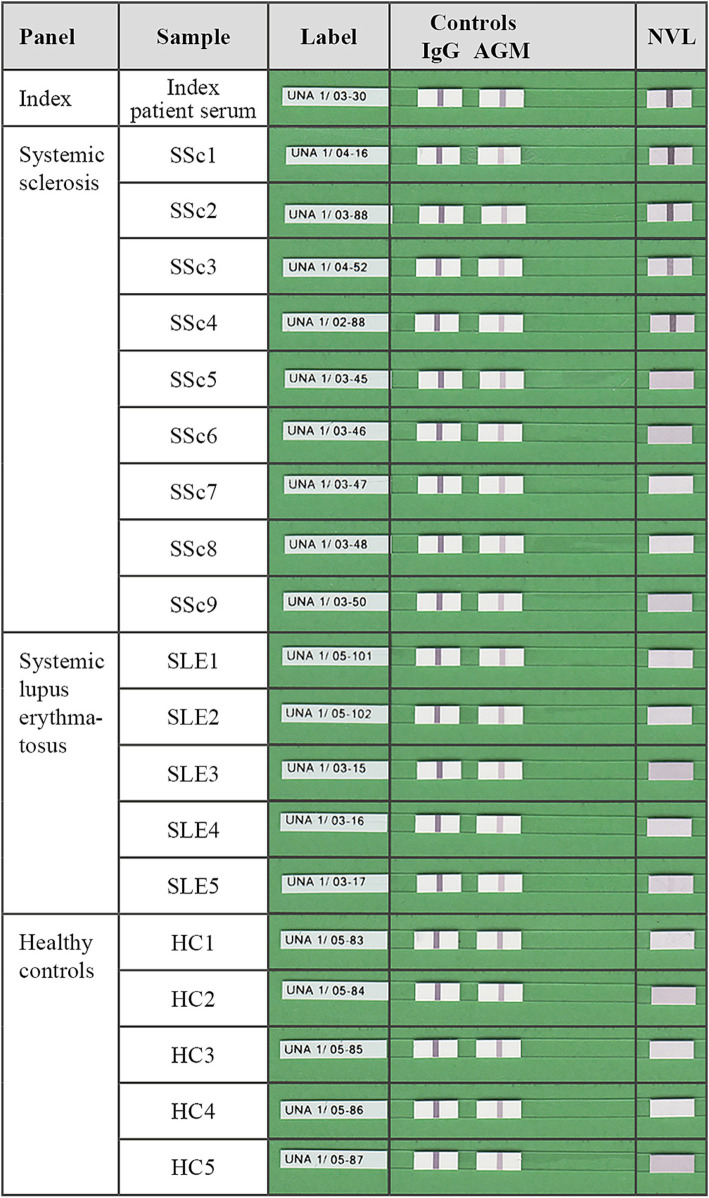
Tabular presentation of representative nuclear valosin-containing-protein-like (NVL) line blot strips after incubation with the anti-NVL-positive index patient serum, four anti-NVL-positive (SSc1-4) and five -negative (SSc5-9) sera from patients with systemic sclerosis, and five samples each of anti-NVL-negative disease controls (SLE1-5) and healthy controls (HC1-5). On each blot strip, the rightmost membrane chip was coated with a line of purified recombinant human NVL that becomes a purple band if a sample contains NVL-specific autoantibodies. Software-based evaluation was used to analyze and interpret the bands’ signal intensity. Correctly performed incubation and correctly used conjugate are indicated by a positive reaction of the serum control band (IgG) and of the conjugate control band (AGM), respectively.

A line blot coated with purified recombinant NVL was developed and validated. Using this assay, the index patient serum showed a positive reaction against NVL, whereas 150 sera from healthy controls did not. Following this, sera from 693 clinically characterized patients with SARD were analyzed with the same line blot, revealing four anti-NVL positive cases (Table 1; Figure 2). NVL-specific reactivity was confirmed using Western blots of purified recombinant NVL (Figure 1F) and recombinant cell-based IFA (RC-IFA) using HEK293 overexpressing NVL. All four anti-NVL positive patients had been diagnosed with SSc, representing 1.1% (4/378, 95% CI, 0.3–2.7%) of the SSc panel (Table 2). The clinical features of these patients are given in Table 3. In two of them, other SSc-relevant AAb were additionally detected (ACA and anti-PM-Scl100) using commercial line blots.

**Table 1:** Anti-NVL reactivity in patient and control sera determined by line blot.

Sample	Intensity NVL line blot [arbitrary units] (positivity cut-off: ≥ 20)
Index patient	107
Systemic sclerosis patient SSc1	105
Systemic sclerosis patient SSc2	97
Systemic sclerosis patient SSc3	55
Systemic sclerosis patient SSc4	87
Other SARD patients, *n* = 689	<20
Healthy controls, *n* = 150	<10

NVL, nuclear valosin-containing-protein-like; SARD, systemic autoimmune rheumatic diseases.

**Table 2:** Prevalence of anti-NVL autoantibodies in SARD patients and healthy controls.

Panel	*n*	Anti-NVL IgG	
		Positive cases	Positivity rate
Systemic sclerosis	378	4	1.1%
Other SARD panels, total	315	0	0%
Systemic lupus erythematosus	165	0	0%
Autoimmune hepatitis	40	0	0%
Rheumatoid arthritis	54	0	0%
Anti-synthetase syndrome	10	0	0%
Myositis	15	0	0%
Primary Sjögren‘s syndrome	11	0	0%
Undifferentiated connective tissue disease	10	0	0%
Mixed connective tissue disease	6	0	0%
Inclusion body myositis	4	0	0%
Healthy controls	150	0	0%

NVL, nuclear valosin-containing-protein-like; SARD, systemic autoimmune rheumatic diseases.

**Table 3:** Demographic and clinical characteristics of anti-NVL-positive patients with systemic sclerosis.

Demographic and clinical characteristics	Anti-NVL positive				
	Patient #1 (SSc1)	Patient #2 (SSc2)	Patient #3 (SSc3)	Patient #4 (SSc4)	Total (*n* = 4)
Sex	F	F	F	F	4 (100%)
Age at diagnosis, years ± SD	48	65	70	58	60 ± 3
SSc diagnosed according to 2013 ACR/EULAR classification criteria^*^	Y	Y	Y	Y	4 (100%)
Cutaneous subtype					
lcSSc	Y	N	Y	Y	3 (75%)
dcSSc	N	Y	N	N	1 (25%)
Raynaud’s phenomenon	Y	Y	Y	Y	4 (100%)
Interstitial lung disease	Y	Y	Y	Y	4 (100%)
Pulmonary arterial hypertension	N	N	N	N	0 (0%)
Arthritis	N	Y	N	N	1 (25%)
Puffy fingers	Y	Y	Y	Y	4 (100%)
Teleangiectasia	Y	Y	Y	Y	4 (100%)
Calcinosis	N	Y	N	Y	2 (50%)
Digital ulcers	Y	N	Y	N	2 (50%)
Scleroderma renal crisis	N	N	N	N	0 (0%)
Gastrointestinal involvement	Y	Y	N	Y	3 (75%)
Sicca symptoms	Y	N	Y	Y	3 (75%)
Primary biliary cholangitis	Y	N	N	N	1 (25%)
Malignancy	N	Y	N	Y	2 (50%)
		Colon cancer	Basalioma	Inflammatory breast	
Autoantibodies					
Anti-NVL	Y	Y	Y	Y	4 (100%)
ACA	N	N	Y	N	1 (25%)
Anti-PM-Scl100	N	N	N	Y	1 (25%)
Anti-Ro-52	Y	Y	Y	Y	4 (100%)

*2013 classification criteria for systemic sclerosis: an American college of rheumatology/European league against rheumatism collaborative initiative (doi: 10.1136/annrheumdis-2013-204424). ACA, anti-centromere antibodies; ACR, American College of Rheumatology; dcSSc, diffuse cutaneous SSc; EULAR, European League Against Rheumatism; F, female; lcSSc, limited cutaneous SSc; N, no; NVL, nuclear valosin-containing-protein-like; SD, standard deviation; SSc, systemic sclerosis; Y, yes.

## 4 Discussion

In this study, NVL was identified as the target of AAb in a patient displaying a high-titer homogenous nucleolar pattern on HEp-2 cells. Using two prototype immunoassays based on the identified autoantigen for serological testing in SARD patients, anti-NVL AAb were detected in 1.1% (4/378, 95% CI: 0.3–2.7%) of SSc patients but not in other disease groups or healthy controls.

As indicated by the name, NVL shows a high level of amino acid similarity to valosin-containing protein (VCP). NVL belongs to ATPases associated with various cellular activities, also referred to as the AAA protein family. The major isoform of NVL (NVL2) contains two tandem AAA domains, a nuclear and a nucleolar localization signal (10). It is ubiquitously expressed, mainly localized in the nucleolus, and involved in the biogenesis of the 60S ribosomal subunit (10). Additionally, it has also essential functions in telomerase biogenesis and pre-mRNA processing (11). It was reported that mutations in NVL may play a role in mental illness, such as major depressive disorder and schizophrenia (12). VCP was previously described as a target of AAb in patients with autoimmune liver diseases and inflammatory myopathies (13, 14). Very recently, Vulsteke et al. identified eight new telomere- and telomerase-associated autoantigens in SSc using immunoprecipitation combined with gel-free liquid chromatography–tandem mass spectrometry (15). They found NVL-specific AAb in two out of 106 patients (1.9, 95% CI: 0.2–6.7%) with SSc, both displaying a nucleolar HEp-2 IFA pattern, which conforms to the findings of the present study. One of the anti-NVL-positive patients was diagnosed with mantle cell lymphoma, which is an interesting finding considering that SSc can be paraneoplastic. Moreover, Perurena-Prieto et al. reported the detection of anti-NVL AAb and homogenous nucleolar HEp-2 cell staining (AC-8) for six out of 307 SSc patients (2.0, 95% CI: 0.7–4.2%) using non-radioactive immunoprecipitation, Western blot assay, and liquid mass spectrometry (16). The clinical characteristics of these patients revealed that anti-NVL AAb are associated with calcinosis and an increased risk of cancer. Together with the four cases we report on, the prevalence of anti-NVL AAb in SSc patients can be assumed to be low (1.1–2.0%). Due to the small number of cases reported to date, it is not yet possible to draw reliable conclusions about clinical associations, necessitating further studies. In addition, the fact that ACA and anti-PM-Scl AAb were found in some anti-NVL-positive SSc patients limits the clinical utility of anti-NVL AAb as a diagnostic marker for SSc. However, in patients with an AC-8 HEp-2 cell pattern who are negative for SSc-specific AAb and other ANAs, testing for anti-NVL AAb may be considered.

In this study, we have developed two prototype monospecific immunoassays, an RC-IFA and a line blot for the detection of anti-NVL AAb in serum. To our knowledge, this is the first study applying a cell-based binding assay for monospecific ANA analysis. Usually, the positive reaction observed in RC-IFA has been described as a specific cytoplasmic reaction in recombinant HEK-cells (17). However, anti-NVL AAb revealed a clear nucleus-specific staining pattern in RC-IFA, which might result from translocation of recombinant NVL into the nucleus due to its nuclear and nucleolar localization signal. RC-IFA has the advantage of presenting the target antigens in a less manipulated condition, providing authentic conformational epitopes and effective conversion of identified novel candidate antigens into immunoassays for further application. Thus, it has a high potential for the identification of other not well-established ANAs. On the other hand, the detection of anti-NVL AAb by line blot offers the possibility of multiplexing NVL with other SARD-associated antigens for testing a broad spectrum of AAb in parallel. As demonstrated by Western blot analysis, NVL-reactive patient sera seem to target linear epitopes. For this reason, line blot testing is a suitable screening method for the detection of anti-NVL AAb. Among the commercially available multiparametric line blots to support the diagnosis of SSc and overlap syndromes, there is currently only one that contains the NVL antigen (EUROLINE Systemic Sclerosis Profile 2, EUROIMMUN). A limitation of this study is that no patients with infections or malignancies were tested by anti-NVL line blot. This limits the clinical application of multiparametric line blots including anti-NVL AAb in the diagnostic work-up of patients with suspected SSc and needs to be addressed in future studies.

Approximately 95% of SSc patients have routinely detectable AAb (1). However, in about 20% of those with a nuclear HEp-2 IFA pattern, the target antigens of these ANAs have not been identified (15). Given the results of this and other studies (15, 16), anti-NVL AAb as biomarkers for SSc may contribute to narrowing this diagnostic gap and might further support the precise diagnosis of patients and personalized treatment. Additional studies are required to determine whether anti-NVL reactivity correlates with a specific SSc phenotype or therapy response.

## Data Availability

Publicly available datasets were analyzed in this study. This data can be found at: UniProt acc. no. O15381-3 (https://www.uniprot.org/uniprotkb/O15381/entry#O15381-3), and GenBank acc. no. BC012105 (https://www.ncbi.nlm.nih.gov/nuccore/BC012105.1/).
