# Supplementary material for: Identification of nuclear valosin-containing-protein-like as a target of anti-nuclear autoantibodies in systemic sclerosis
Source: Front Med (Lausanne). 2025 Jan 21;11:1477365. doi: 10.3389/fmed.2024.1477365 (PMC11790567; doi:10.3389/fmed.2024.1477365)
Supplement: Supplementary file 1 [file Table_1.docx]

Supplementary Material

**Supplementary Table 1** | Demographics and diagnostic criteria of the study population.

| **Characteristics** | **Systemic sclerosis** | **Systemic lupus erythe-matosus** | **Auto-immune hepatitis*** | **Rheumatoid arthritis** | **Anti-synthetase syndrome** | **Myositis** | **Primary Syögren’s syndrome** | **Undiff. connective tissue disease** | **Mixed connective tissue disease** | **Inclusion body myositis** | **Healthy controls** |
| --- | --- | --- | --- | --- | --- | --- | --- | --- | --- | --- | --- |
| Total number, n | 378 | 165 | 40 | 54 | 10 | 15 | 11 | 10 | 6 | 4 | 150 |
| Mean age ± SD (range) [ND], years | 57 ± 15  (19-87) | 47 ± 15  (17-83) [14] | 14 ± 3  (6-18) | 61 ± 16  (30-87) | 60 ± 14  (33-82) | 58 ± 14  (35-78) | 70 ± 9  (57-80) | 55 ± 16  (29-81) | 51 ± 17  (34-82) | 76 ± 5  (72-83) | 41 ± 13  (19-67) |
| Female:male [ND], n | 318:59 [1] | 126:25 [14] | 17:23 | 41:13 | 9:1 | 9:6 | 9:2 | 6:4 | 6:0 | 1:3 | 18:114 [18] |
| Ethnicity, n |  |  |  |  |  |  |  |  |  |  |  |
| African American | 1 | 11 | ― | ― | ― | ― | ― | ― | ― | ― | ― |
| Asian | 1 | 20 | ― | ― | ― | 2 | ― | 1 | ― | ― | ― |
| Middle Eastern | 2 | 1 | ― | ― | ― | ― | ― | ― | ― | ― | ― |
| Hispanic | ― | 20 | ― | ― | ― | ― | ― | ― | ― | ― | ― |
| White | 374 | 29 | ― | 24 | 10 | 13 | 11 | 9 | 6 | 4 | ― |
| Unknown | ― | 84 | 40 | 30 | ― | ― | ― | ― | ― | ― | 150 |
| Diagnostic criteria as published in | [2013 ACR/ EULAR classification criteria for SSc](https://doi.org/10.1136/annrheumdis-2013-204424) | [1997 Update of ACR revised classification criteria for SLE](https://onlinelibrary.wiley.com/doi/abs/10.1002/art.1780400928?sid=nlm%3Apubmed) | [2015 EASL clinical practice guidelines for AIH](https://doi.org/10.1016/j.jhep.2015.06.030) | [2010 ACR/ EULAR classification criteria for RA](https://academic.oup.com/rheumatology/article-abstract/51/suppl_6/vi5/1787592?redirectedFrom=fulltext&login=false) | [2016 Diagnosis and treatment of anti-synthetase syndrome](https://doi.org/10.1097/cpm.0000000000000171) | [2017 EULAR/ ACR classification criteria for JIIM](https://acrjournals.onlinelibrary.wiley.com/doi/10.1002/art.40320) | [2016 ACR/ EULAR classification criteria for Sjögren’s syndrome](https://acrjournals.onlinelibrary.wiley.com/doi/10.1002/art.39859) | [2014 Diagnosis and classification of UCTD](https://doi.org/10.1016/j.jaut.2014.01.019) | [1987 Classification and diagnostic criteria for MCTD](https://www.researchgate.net/publication/312909486_Classification_and_diagnostic_criteria_for_mixed_connective_tissue_disease) | [2013 ENMC diagnostic criteria for IBM](https://www.sciencedirect.com/science/article/abs/pii/S0960896613009504) | ― |

*Sera from adult and elderly patients with autoimmune hepatitis were not available for testing. Therefore, only children and adolescents with autoimmune hepatitis were included in this group, leading to a significantly lower mean age compared to the other study groups.

*ND, no data; SD, standard deviation*

**Supplementary Table 2** | Protein identified using matrix-assisted laser desorption/ionization-time of flight mass spectrometry by peptide mass fingerprinting after immunoprecipitation from HEp-2 cells with index patient serum.

| **Protein name (UniProt)** | **Accession number** | **Cut-off** | **Mascot Score** | **Sequence coverage (%)** | **Peptides** | **Protein MW (Da)** |
| --- | --- | --- | --- | --- | --- | --- |
| Nuclear valosin-containing protein-like,  OS = *Homo sapiens*, GN = NVL, PE = 1, SV = 1 | O15381 | 59 | 164 | 33 | 21 | 96,017 |

Data base: SwissProt (taxonomy *Homo sapiens*)

*Da, Dalton; GN, gene name; MW, molecular weight; OS, organism species; PE, protein existence; SV, sequence version*
